# Methotrexate for the Treatment of Thyroid Eye Disease

**DOI:** 10.1155/2014/128903

**Published:** 2014-01-08

**Authors:** Diego Strianese, Adriana Iuliano, Mariantonia Ferrara, Chiara Comune, Immacolata Baronissi, Pasquale Napolitano, Alessia D'Alessandro, Piergiacomo Grassi, Giulio Bonavolontà, Paola Bonavolontà, Antonio Sinisi, Fausto Tranfa

**Affiliations:** ^1^Department of Neuroscience, Odontostomatological and Reproductive Sciences, University “Federico II” of Naples, Via Pansini No. 5, 80131 Naples, Italy; ^2^Department of Endocrinology, Second University of Naples, Italy

## Abstract

*Background/Aim*. To evaluate the efficacy of methotrexate for the treatment of thyroid eye disease (TED). *Methods*. 36 consecutive patients with active TED, previously treated with corticosteroids but stopped due to the occurrence of side effects, were commenced on methotrexate therapy. Two different weekly doses were administered depending on the weight of the patient (7.5 mg or 10 mg). Clinical activity score (7-CAS), visual acuity (VA), ocular motility, exophthalmos, and eyelid position were retrospectively evaluated at 3, 6, and 12 months and compared with baseline data. *Results*. There was a statistically significant improvement in 7-CAS at 3, 6, and 12 months after treatment (*P* < 0.0001). There was no significant change in visual acuity. Ocular motility disturbances improved at 6 and 12 months (*P* < 0.001). There was no significant change in exophthalmos (mean 24 mm, SD 3 mm) or eyelid position (marginal reflex distance mean 6 mm, SD 1.5 mm) during the follow-up period. No side effects were registered. *Conclusions*. Methotrexate therapy is effective in reducing CAS and ocular motility disturbances. No significant improvement in proptosis or eyelid retraction should be expected from this treatment. Eventually, it might be considered a suitable alternative treatment in TED for patients who cannot tolerate steroids.

## 1. Introduction

Thyroid eye disease (TED) is an autoimmune disease involving the retroocular tissues associated with Graves' disease [1, 2]. Typical signs and symptoms include proptosis, retroorbital pain, tearing, conjunctival redness and edema, corneal lesions, impaired extraocular motility with or without diplopia, periorbital edema, visual impairment, and, rarely, blindness.

Treatment options for TED include immunosuppressive agents, radiotherapy, and various surgical procedures such as orbital decompression, squint surgery, and correction of eyelid retraction [3–5].

Glucocorticoids are still the most widely used immunosuppressive agents for the treatment of TED and appear to be the most effective for associated soft tissue inflammation, optic neuropathy, and extraocular muscle impairment [6, 7]. The main disadvantages of glucocorticoid therapy are the potential recurrence of the disease after discontinuation and the side effects in long-term treatment [7]. Several alternative therapies have been proposed to manage resistant TED such as orbital radiation therapy, several other immunosuppressive agents, and biological drugs. However, the effectiveness of these treatments is still widely debated in the literature [8–15].

The aim of this study is to evaluate the efficacy of methotrexate used as single therapy in a group of patients with recurrence of the active TED after withdrawal of corticosteroids due to the onset of side-effects.

## 2. Materials and Methods

This is a retrospective comparative case series of 36 patients, from 2001 to 2010, who experienced reactivation of TED after withdrawal of corticosteroid therapy because of side-effects. The study followed the Declaration of Helsinki on medical protocol and ethics.

Patients with sight threatening for compressive optic neuropathy were excluded from this study as they needed urgent treatment such as high-dose steroids or surgical decompression to prevent optic atrophy.

Before methotrexate therapy, all patients received as initial treatment intravenous pulse-methylprednisolone therapy at a dose of 10 mg/kg twice a week for 6 weeks (on Tuesdays and Thursdays), followed by oral prednisone at a dose of 30 mg/day, tapered to discontinuation over approximately 2 months. Patients with recurrence of inflammatory signs (7-point clinical activity score (7-CAS) ≥ 3 and in patients with asymmetric presentation the eye with the worse score was considered [16]) received as maintenance oral prednisone at a dose of 10 mg/day. From this group, 36 patients who stopped corticosteroids due to the occurrence of side-effects or intolerance to maintenance steroid therapy were commenced on methotrexate therapy. During a time period of 9 years, 36 patients received methotrexate as an alternative therapy to steroids. Reasons for cessation of steroid therapy included mood swings (25% of patients), hyperglycemia (21% of patients), hypertension (19% of patients), fecal blood (7% of patients), and complaining of puffiness, sleeplessness, facial flushing, slight tremor, and agitation (28% of patients). Length of previous corticosteroid therapy ranged from 2 months to 1 year (median 5.12 months, Table 1). Time between pulse therapy and second-line treatment ranged from 2 months to 1 year (median 8 months, Table 1).

There were 11 male patients and 25 female patients. Twenty-four (67%) were smokers. Patients were aged from 35 to 70 years with a mean age of 52 years (Table 1). At the onset of TED, hyperthyroidism was treated in 20 patients with thiamazole, 8 with a combination of thiamazole and thyroidectomy, 3 with radioiodine therapy, and 5 with thiamazole and radioiodine (Table 1). Thyroid function at time of treatment with methotrexate was as follows: 22 patients (61%) were euthyroid (12 still using thiamazole and 10 without treatment), 3 patients (8%) were still hyperthyroid (using thiamazole), and 11 patients (30%) were hypothyroid (using levothyroxine).

### 2.1. Methotrexate Therapy

Patients who constituted the study group received methotrexate therapy for 1 year.

Pretreatment laboratory investigations included complete blood count; assessment of liver, kidney, and lung functions; serum immunoglobulin levels; and hepatitis B and C serology.

Methotrexate was administered at two different weekly doses depending on the weight of the patient: 7.5 mg for patients weighing less than 60 kg and 10 mg for those weighing more than 60 kg.

The weekly dose of 7.5 mg was orally administered and fractionated as follows: two tablets of 2.5 mg on Thursday and one on Friday, while the dose of 10 mg was administered and fractionated as three tablets of 2.5 mg on Thursday and one on Friday. To minimize the potential toxicity, a dose of 15 mg of folic acid was prescribed for each patient on Sundays. Laboratory evaluation including a complete blood count and liver enzyme testing was performed every 2 weeks.

### 2.2. Examination

The following parameters were measured at baseline (premethotrexate treatment) and at 3, 6, and 12 months after commencement of methotrexate treatment: 7-CAS [17, 18], ocular motility, exophthalmos, eyelid aperture, and visual acuity (VA). 7-CAS ≥ 3 defined active TED. Ocular motility was measured using the Light Reflex Method, recently validated for TED motility evaluation [19]: a score of 45° was given when the light's reflex was at the limbus, 30° at half way between the limbus and pupil edge and 15° at the pupil edge. Improvement in ocular motility was considered if variation was at least of 15°, as previously reported [19]. Exophthalmos was measured with Hertel exophthalmometry: a decrease of 2 mm was considered significant. Eyelid retraction was evaluated by measuring the Margin Reflex Distance (MRD). VA was measured with the Snellen Chart. Where different scores or measures of severity existed for the two separate eyes of one individual, scores of the worse eye were considered for analysis.

### 2.3. Statistical Analysis

Mean 7-CAS, exophthalmometry, and MRD measurements were compared before and after methotrexate treatment using Student's *t*-test. The chi squared test (*χ* ^2^) was used to compare the results of VA and ocular motility data. MedCalc statistical software, version 11.3.0.0, was used for all statistical analyses. Only values of *P* ≤ 0.001 were considered to be statistically significant.

## 3. Results

### 3.1. Outcome Parameters

#### 3.1.1. 7−CAS

Mean 7-CAS score before methotrexate therapy was 4.05 (SD 1.11). There was a significant improvement in 7-CAS score at 3, 6, and 12 months (*P* < 0.0001) (Table 2). At 3 months, 24 patients (67%) had a reduction of 7-CAS in both eyes; at 6 months, 29 patients (80%) had a reduction of 7-CAS in both eyes; at 12 months, 34 patients (94%) had a reduction of 7-CAS in both eyes.

#### 3.1.2. Visual Acuity (VA)

Before methotrexate therapy, 18 patients (50%) had a VA of 20/20, 8 patients (22%) had 20/25, 8 patients (22%) had 20/30, and 2 patients (6%) had 20/50. After 3 months there were no changes in VA. After 6 months, 2 patients had an improvement of one line on the Snellen Chart. After 12 months of followup only 1 patient had an improvement of three lines on the Snellen Chart. There were no significant improvements in VA with treatment.

#### 3.1.3. Ocular Motility

15 patients (42%) had some degree of gaze restriction at baseline. At 3 months, 4 patients improved (27% of patients with ocular motility disturbances), and at 6 and 12 months a total of 10 patients improved (67% of patients with ocular motility disturbances). The reduction of ocular motility restriction after 6 and 12 months of treatment was significant (*P* < 0.001) (Table 3).

#### 3.1.4. Exophthalmos

Mean Hertel exophthalmometry measurement was 24 mm (range from 17 to 27, SD 3). There was no significant change at 3, 6, and 12 months after treatment (Table 3).

#### 3.1.5. MRD

Mean MRD was 6 mm (range from 4 to 7, SD 1.5). There was no significant change at 3, 6, and 12 months after treatment (Table 3).

Methotrexate was well tolerated in all patients and no side effects were registered.

## 4. Discussion

The biological process of TED has originally been described by Rundle's curve [20]. The orbital disease usually starts with a dynamic period of active inflammation characterized by pain, redness, and swelling of the eyelids. During the active phase, disease severity increases and then plateaus. After this, disease severity slowly decreases until stable end stage, called inactive phase. Although the shape of the curve is representative of disease course for majority of patients, the time axis of Rundle's curve shows high individual variability: it can take from a few months to more than 5 years to reach the static phase. Prompt restoration of stable euthyroidism and immunosuppression may decrease the duration of the dynamic phase and reverse the tendency to progression.

Management of active inflammation in TED in the present study was based on the 7-CAS. When the inflammatory grade was 3 or greater, or if there was evidence of progression in the inflammation, immunosuppressive treatment was necessary. Systemic steroids are generally considered first-line treatment for TED as they can be given with systemic (oral or intravenous) or local (subconjunctival or retrobulbar) administration [5]. The efficacy of high-dose intravenous corticosteroid pulse therapy has been widely reported [6]. In our study all patients initially received corticosteroid pulse therapy, which is routinely used in our unit for patients with 7-CAS higher than 3. For those patients with persistent TED or reactivation after pulse therapy, long-term corticosteroid therapy is commonly used. However long-term use of corticosteroid can lead to complications such as high blood pressure, Cushing syndrome, diabetes, osteoporosis, fragility fractures, and in rare cases acute hepatitis [20]. Patients included in this study developed such side-effects over the treatment course, and when therapy was withdrawn, they experienced an increase in the inflammation score.

Many alternative therapies have been reported for such patients, although their efficacy is still under debate [12, 14, 15]. The efficacy of methotrexate has been reported for many autoimmune diseases requiring a long-term maintenance therapy such as rheumatoid arthritis, inflammatory bowel disease, and Wegener's granulomatosis [21]. Methotrexate is an immunosuppressive drug that inhibits dihydrofolate reductase enzyme, leading to the inhibition of the DNA, RNA, and protein synthesis [15, 22]. Cytotoxic and antiproliferative effects can be seen at high doses, while anti-inflammatory and immunomodulatory effects can be observed with chronic low-dose treatment [23, 24]. The use of methotrexate is proposed on the basis of the autoimmune nature of TED [11, 15, 25, 26].

Patients who received methotrexate showed an early significant improvement of 7-CAS at 3, 6, and 12 months (*P* < 0.0001). Although none of patients included in this study had an optic neuropathy, VA improvement was reported but it was not significant. The improvement in ocular motility restriction was statistically significant after 6 and 12 months of methotrexate treatment (*P* < 0.001). Exophthalmometry and MRD measurements showed no statistically significant differences before and after treatment. Serious adverse effects were not reported in our study. However, side effects such as gastrointestinal disturbances, increased risk of opportunistic infections, bone marrow depression, interstitial pneumonitis, liver toxicity, asthenia, and fecal occult blood should always be considered since they have been reported in the literature [27, 28].

The addition of folate may reduce methotrexate toxicity, without affecting its effectiveness [14, 23]. The glucocorticoid-sparing effect of methotrexate is confirmed in our study, but this drug should not be used for more than 2 years in order to avoid the risk of megaloblastic anemia and lymphomas [22].

All the participants into this study had experienced recurrence of inflammatory signs of TED after withdrawal of corticosteroid treatment such as ocular pain and discomfort due to chemosis, hyperemia, and eyelid edema. These patients, as reported in previous studies, seem to have a phase of activity longer than usual [29]. Early surgical decompression could be considered for these patients even though its effectiveness needs to be confirmed [30]. In addition, patients with this condition may refuse surgery and methotrexate may be offered to them as a second-line therapy in order to contain the inflammatory process and prevent further complications due to fibrosis process. Hence, the indication for treatment with methotrexate should be a recurrent TED with 7-CAS ≥ 3 after withdrawal of steroids because of onset of steroid side-effects or intolerance.

Our study suggests that methotrexate therapy is useful for reducing the signs and symptoms of inflammation in TED, shortening the active phase, and reducing eye discomfort since all patients had a statistically significant reduction in inflammatory score. Methotrexate appears to be a suitable second-line treatment for patients with recalcitrant TED to control the clinical profile and delay surgery until the disease stability.

## Figures and Tables

**Table 1:** Characteristics of enrolled patients (*n* = 36).

Characteristics	Value
Mean age (years)	52 (35–70)
Males (no.)	11
Females (no.)	25
Smokers (no.)	24
Time from the onset of ocular symptoms to pulse steroid therapy	5.12 months (2 m–1 y)
Median time between pulse therapy and second-line treatment	8 months (2 m–12 m)

**Table 2:** 7-CAS results before and after 3, 6, and 12 months of methotrexate therapy.

	Baseline	At 3 months	At 6 months	At 12 months
Mean 7-CAS score ± SD	4.05 ± 1.112	2.50 ± 1.079	2.23 ± 1.079	2.16 ± 1.098
*P*=		**<0.0001**	**<0.0001**	**<0.0001**

Bold data are referred to statistically significant *P* values.

**Table 3:** Ocular motility, exophthalmos, and MRD results before and after 3, 6, and 12 months of methotrexate therapy.

Parameters	Baseline	At 3 months	At 6 months	At 12 months
Ocular motility Patients with gaze restriction (no. and %)	15 (42%)	11 (30%)	5 (14%)	5 (14%)
*P* value *P* (versus baseline)		=0.03	** <0.001**	**<0.001**
Mean exophthalmometry (mm) ± SD	24 ± 3	24 ± 2.5	24 ± 2	24 ± 3.5
*P* value *P* (versus baseline)		=1	=0.1	=0.03
Mean MRD (mm) ± SD	6 ± 1.5	6 ± 1	5 ± 1.5	5 ± 1.5
*P* value *P* (versus baseline)		=1	=0.06	=0.06

Bold data are referred to statistically significant *P* values.

## References

[1] Bahn RS (2010). Graves’ ophthalmopathy. *The New England Journal of Medicine*.

[2] Comerci M, Elefante A, Strianese D (2013). Semiautomatic regional segmentation to measure orbital fat volumes in thyroid-associated ophthalmopathy. Avalidation study. *The Neuroradiology Journal*.

[3] Uccello G, Vassallo P, Strianese D, Bonavolonta G (1994). Free levator complex recession in Graves’ ophthalmopathy. Our experience. *Orbit*.

[4] Koshiyama H, Koh T, Fujiwara K, Hayakawa K, Shimbo S-I, Misaki T (1994). Therapy of Graves’ ophthalmopathy with intravenous high-dose steroid followed by orbital irradiation. *Thyroid*.

[5] Wiersinga WM, Prummel MF (2002). Graves’ ophthalmopathy: a rational approach to treatment. *Trends in Endocrinology and Metabolism*.

[6] Bartalena L, Marcocci C, Tanda M, Pinchera A (2002). Management of thyroid eye disease. *European Journal of Nuclear Medicine*.

[7] Krassas GE, Heufelder AE (2001). Immunosuppressive therapy in patients with thyroid eye disease: an overview of current concepts. *European Journal of Endocrinology*.

[8] Marcocci C, Kahaly GJ, Krassas GE (2011). Selenium and the course of mild Graves’ orbitopathy. *The New England Journal of Medicine*.

[9] Mourits MP, Van Kempen-Harteveld ML, García García MB, Koppeschaar HPF, Tick L, Terwee CB (2000). Radiotherapy for Graves’ orbitopathy: randomised placebo-controlled study. *The Lancet*.

[10] Minakarau N, Ezra DG (2013). Rituximab for thyroid-associated ophthalmopathy. *Cochrane Database of Systematic Reviews*.

[11] Sanyal P, Bing-You RG, Braverman LE (2008). Use of methotrexate to treat isolated graves ophthalmopathy developing years after thyroidectomy and iodine 131 treatment of papillary thyroid cancer. *Endocrine Practice*.

[12] Bartalena L, Marcocci C, Tanda ML (2002). Orbital radiotherapy for Graves’ ophthalmopathy. *Thyroid*.

[13] Prummel MF, Berghout A, Wiersinga WM, Mourits MP, Koornneef L, Blank L (1993). Randomised double-blind trial of prednisone versus radiotherapy in Graves’ ophthalmopathy. *The Lancet*.

[14] Smith JR, Rosenbaum JT (2001). A role for methotrexate in the management of non-infectious orbital inflammatory disease. *British Journal of Ophthalmology*.

[15] Bartalena L, Tanda ML, Medea A (2002). Novel approaches to the management of Graves’ ophtalmopathy. *Hormones*.

[16] Strianese D, Piscopo R, Elefante A (2013). Unilateral proptosis in thyroid eye disease with subsequent contralateral involvement: retrospective follow-up study. *BMC Ophthalmology*.

[17] Mourits MP, Prummel MF, Wiersinga WM, Koornneef L (1997). Clinical activity score as a guide in the management of patients with Graves’ ophthalmopathy. *Clinical Endocrinology*.

[18] Pinchera A, Wiersinga W, Glinoer D (1992). Classification of eye changes of Graves’ disease. *Thyroid*.

[19] Dolman PJ, Cahill K, Czyz CN (2012). Reliability of estimating ductions in thyroid eye disease: an international thyroid eye disease society multicenter study. *Ophthalmology*.

[20] Rundie FF (1945). Development and course of exophthalmos and ophthalmoplegia in Graves’ diseasee with special reference to the effect of thyroidectomy. *Clinical Science*.

[21] Songsiridej N, Furst DE (1990). Methotrexate—the rapidly acting drug. *Bailliere's Clinical Rheumatology*.

[22] Le Moli R, Baldeschi L, Saeed P, Regensburg N, Mourits MP, Wiersinga WM (2007). Determinants of liver damage associated with intravenous methylprednisolone pulse therapy in Graves’ ophthalmopathy. *Thyroid*.

[23] Seitz M (1999). Molecular and cellular effects of methotrexate. *Current Opinion in Rheumatology*.

[24] Rampton DS (2001). Methotrexate in Crohn’s disease. *Gut*.

[25] Bartalena L, Pinchera A, Marcocci C (2000). Management of graves’ ophthalmopathy: reality and perspectives. *Endocrine Reviews*.

[26] Alarcón GS, Kremer JM, Macaluso M (1997). Risk factors for methotrexate-induced lung injury in patients with rheumatoid arthritis: a multicenter, case-control study. *Annals of Internal Medicine*.

[27] D’Andrea N, Triolo L, Margagnoni G, Aratari A, Sanguinetti CM (2010). Methotrexate-induced pneumonitis in Crohn’s disease: case report and review of the literature. *Multidisciplinary Respiratory Medicine*.

[28] Amital H, Arnson Y, Chodick G, Shalev V (2009). Hepatotoxicity rates do not differ in patients with rheumatoid arthritis and psoriasis treated with methotrexate. *Rheumatology*.

[29] Menconi F, Profilo MA, Leo M (2013). Spontaneous improvement of untreated mild Graves’ ophthalmopathy: the Rundle curve revisited. *Thyroid*.

[30] Baldeschi L, Macandie K, Koetsier E, Blank LECM, Wiersinga WM (2008). The influence of previous orbital irradiation on the outcome of rehabilitative decompression surgery in graves orbitopathy. *American Journal of Ophthalmology*.

